# Membrane Dynamics Regulated by Cytoskeleton in Plant Immunity

**DOI:** 10.3390/ijms24076059

**Published:** 2023-03-23

**Authors:** Yuqing Lu, Yuan Zhang, Na Lian, Xiaojuan Li

**Affiliations:** 1 Key Laboratory of Genetics and Breeding in Forest Trees and Ornamental Plants, Ministry of Education, College of Biological Sciences and Technology, Beijing Forestry University, Beijing 100083, China; 2 Institute of Tree Development and Genome Editing, College of Biological Sciences and Technology, Beijing Forestry University, Beijing 100083, China

**Keywords:** cytoskeleton, endocytosis and exocytosis, plant immunity, plasma membrane protein

## Abstract

The plasma membrane (PM), which is composed of a lipid layer implanted with proteins, has diverse functions in plant responses to environmental triggers. The heterogenous dynamics of lipids and proteins in the plasma membrane play important roles in regulating cellular activities with an intricate pathway that orchestrates reception, signal transduction and appropriate response in the plant immune system. In the process of the plasma membrane participating in defense responses, the cytoskeletal elements have important functions in a variety of ways, including regulation of protein and lipid dynamics as well as vesicle trafficking. In this review, we summarized how the plasma membrane contributed to plant immunity and focused on the dynamic process of cytoskeleton regulation of endocytosis and exocytosis and propose future research directions.

## 1. Introduction

In order to survive and reproduce, plants have evolved two efficient immune systems. When plants are attacked by pathogens, plants use pattern recognition receptors (PRRs) on the surface of the cytoplasmic membrane to recognize microbial/pathogen-associated molecular patterns (MAMPs/PAMPs) or host-derived damage-associated molecular patterns (DAMPs) to further activate the first immune system called pattern-triggered immunity (PTI) [1,2,3]. For successful infection and colonization, pathogens secrete effectors into plant cells that interfere with host physiology and inhibit PTI [4]. The second layer of immune barrier activation is related to disease intracellular immune receptors, which belong to the nucleotide-binding leucine-rich repeat (NLRs) class and trigger immunity by identifying effector factors. Therefore, the mechanism of effector–host recognition is named effector-triggered immunity (ETI) [4]. Although PTI and ETI are involved in the activation of two unique types of receptors, the downstream immune outputs are strikingly similar [5,6]. It should be noted that these overlapping immune outputs include changes in Ca^2+^ flux, rapid bursts of reactive oxygen species (ROS), mitogen-activated protein kinase (MAPK) cascades, transcriptional reprogramming and phytohormone signaling, indicating the junction and convergence of these two signaling cascades [7]. Furthermore, ETI is not a separate immune pathway which relies on the PTI machinery to function effectively [8]. Roux et al. [9] reported that the co-receptors BAK1 and BKK1 in PTI are also required for ETI response against *Hyaloperonospora arabidopsidis* (*Hpa*), suggesting PTI and ETI share central components of these two systems; the two immune responses combine to promote a strong immunity. Though growing evidence points to the presence of complex interactions between PRR- and NLR-mediated signaling cascades [10], their connection is unknown.

Plant immunity is closely associated with the cell cytoskeleton. The plant cytoskeleton is composed of microfilaments (MFs) and microtubules (MTs). MFs, also known as the actin cytoskeleton, are generated by polymerizing globular (G)-actin into filamentous (F)-actin [11]. MTs are made up of a complicated array of α/β-tubulin heterodimers [12]. The cytoskeleton plays an important role in the process of disease resistance, especially the actin cytoskeleton, which is a signal transduction platform in the plant immune process. The increase in actin filament density is one of the conserved PTI responses [13]. After MAMP or DAMP treatment, the actin filament density increased significantly at the infection site [14]. Moreover, fungal invasion of plants triggers MF and MT reorganization, leading actin filaments to form radial microfilament bundles, which are conducive to the transport of organelles and vesicles to the invasion site and enhance the resistance of pathogenic fungal invasion [15]. When the host plant is infected by powdery mildew, host nucleus, ER and Golgi accumulation occur concurrently with fast reorganization of actin filaments [15]. The use of actin polymerization inhibitor latrunculin B (Lat B) to inhibit the actin filaments aggregation affects penetration resistance, causing plants to increase the susceptibility of pathogenic bacteria [13]. In addition, the cytoskeleton regulates callose deposition in the PTI immune response. Yang et al. [15] have reported that inactivation of myosin by pharmacological inhibitors prevents deposition of callose into the apoplastic papillae, resulting in the attenuation of penetration resistance. The translocation of callose synthases to the PM requires both actin filaments and MT, and the disruption of either cytoskeletal network causes callose synthases to dysfunction [16]. The cytoskeleton plays a positive regulatory role in plant immunity, actively responding to pathogenic signals and rapidly changing its arrangement to resist pathogenic infection.

The structures of the plant cytoskeleton are highly dynamic and precisely regulated, which have been studied and summarized in some wonderful reviews [17,18]. For instance, Mao et al. [19] summarized the key regulators and dynamics of cytoskeleton reorganization in response to environmental stress. Li et al. [20] discuss the array changes of MF and MT cytoskeletons during the process of the plant cell cycle. The high-resolution three-dimensional structure of MF in pollen was recently resolved by cryo-electron microscopy [21]. In this review, we will focus on the role of the cytoskeleton in regulating protein dynamics, membrane lipid composition and vesicle transport, and we summarize the latest research developments. Finally, we also provide some prospects for future development.

## 2. Membrane Lipids and Proteins Involved in Plant Immunity

The plasma membrane provides a natural barrier for the cell, which is composed of lipids and proteins. Almost all the important functions of the cell are related to the plasma membrane, which not only protects the cell but also participates in many physiological processes such as cell signal recognition and transmission, material transportation, etc. [10]. The plasma membrane plays a key role during pathogen attack. It is involved in pathogen recognition and the transmission of external signals into the downstream of the cell, which further helps to regulate plant immunity responses.

### 2.1. Functional Diversity of the Membrane Lipids That Are Involved in Immunity Response

Membrane lipids include phospholipids and sterols in higher plants, and the constituents play a critical role in maintaining the stability of the cell membrane structure and function. Phospholipids, which consist of glycerophospholipids and sphingolipids, are abundant in all plasma membranes. Glycerophospholipids are widely conserved in animals and plants, including phosphatidylcholine (PC), phosphatidylethanolamine (PE), phosphatidylserine (PS), phosphatidylinositol (PI) and diacylglycerols (DAGs). Phospholipid-based signal transduction has been reported to be involved in plant immune responses. For example, phospholipase D can be hydrolyzed to generate phosphatidic acid, which serves the function of second messenger to activate ROS to trigger actin rearrangements [14].

Sphingolipids, which account for up to 40% of total lipids in plants [22], can be grouped into four classes in plants: free long-chain bases (LCBs); ceramides (CERs), comprising LCBs with fatty acids (FA); glucosylceramides; and glycosyl inositol phosphoryl ceramides (GIPCs) [23]. The 2-hydroxylation of acyl chains in plant sphingolipids are a typical hydroxylation modification, which are important in stress responses. A recent study showed that 2-hydroxy sphingolipids contribute to the formation of plasma membrane lipid rafts [24]. The formation of plasma membrane domains also requires the aggregation of sterols. It has been identified that plants sterols contain around 250 different types of sterols and sterol conjugates, including free sterols, sterol esters, sterol glycosides and acyl sterol glycosides [25]. Particularly, sterol glycosides and acyl sterol glycosides gather in lipid rafts [26].

The lipid raft/membrane raft is a dynamic domain of a liquid-ordered phase with a diameter of 10 to 200 nm, which is characterized by tight lipid filling [22]. It is reported that these regions are resistant to extraction by non-ionic detergents at low temperatures, so they are also known as detergent-resistant membranes (DRMs) [27]. A variety of physiological and biochemical reactions in cells take place on membrane raft microdomains, which are considered as the platform that recruit multiple immune molecules to participate in the defense response. Moreover, Cui et al. [28] reported that flagellin sensing2 (FLS2) protein was localized to the microdomain through oligomerization after being stimulated by flg22. A similar pattern of results was obtained in the study of Remorin1.3 (REM1.3) [29].

### 2.2. Functional Diversity of the Membrane Proteins That Are Involved in Immunity Response

Membrane proteins are important executors of plasma membrane function. When plants are attacked by pathogens, plants employ PRRs to recognize specific chemical components of invading pathogenic microorganisms and activate the plant immune system to make an immune response. Most PRRs contain an extracellular domain with leucine-rich repeats (LRRs), an intracellular kinase domain and a single-pass transmembrane domain. At present, the well-studied PRRs include FLS2, elongation factor Tu receptor (EFR), chitin elicitor receptor kinase 1 (CERK1) and PEP Receptors (PEPRs) [30]. PRR is the immune switch of PTI, and the first PRR identified in plants was *Arabidopsis* FLS2. After the extracellular LRR domain of FLS2 recognizes bacterial flagellin, FLS2 interacts with the co-receptor BAK1 to form a stable heterodimer [31,32]. Similar to FLS2, the EFR recognizes the EF-Tu and elf18 peptides and forms EFR–BAK1 complexes to activate plant immunity [33]. The perception of PEP by a plant elicitor peptide receptor (PEPR) leads to the fluctuation of Ca^2+^ to trigger the downstream immune signal [34]. Importantly, PRRs can also bind effectors, including FLS2 and EFR, to hinder immune responses. For example, FLS2 can directly interact with *Pseudomonas syringae* effector AvrPto to inhibit the kinase activity of FLS2 [35].

In addition, NLRs on the plasma membrane have been reported to recognize effectors, which are secreted by pathogens, directly or indirectly. Plant NLRs contain three domains: an N-terminal variable domain, a middle nucleotide binding domain and a C-terminal LRR domain [36]. NLRs are classified into two groups that are defined by different N-terminal domains: the coiled-coil-type NLRs (CNLs) and Toll/interleukin-1 receptor/resistance protein type NLRs (TNLs) [37]. After immune activation, NLRs often form oligomeric complexes, which are called resistosomes in plants. For instance, CNL receptor hopz-activated resistance 1 (ZAR1) in *Arabidopsis* indirectly recognizes the effector AvrAC through bait protein PBL2. AvrAC uridylylates the PBL2, which is then recruited to form a ZAR1–RKS1–PBL2^UMP^ complex [38]. Further work proved that ZAR1-activated resistosome serves as a calcium-permeable cation channel to initiate immunity response, such as production of ROS and cell death [39]. Another typical representative of TNL resistance protein is *Arabidopsis thaliana* recognition of peronospora parasitica1 (RPP1). RPP1 directly combines with *Hpa* effector ATR1 to form tetrameric resistosome, which promotes the NADase hydrolytic activity to activate downstream EDS1 and NRG1 immune pathways [40,41].

Besides receptors, other membrane proteins have also been reported to participate in the defense response, such as respiratory burst homologs (RBOHs) and plasma membrane intrinsic proteins (PIPs). ROS produced via RBOH appear to cause local and/or systemic reprogramming in order to activate innate immunity [42]. Studies about leaf microbiota show that RBOHD and RBOHF maintain the homeostasis of the microbiota to prevent imbalances [43]. PIPs not only transport water but also extend to subcellular transport of ROS, such as H_2_O_2_ [44]. Studies have shown that AtPIP2;1 was phosphorylated by BAK1 during the response of guard cells to flg22 and increased the transport rate of water and H_2_O_2_, thereby regulating the closure of stomata to achieve an immune response [45]. Interestingly, due to the three extracellular regions of PIPs that are exposed to the extracellular environment, plant pathogenic bacteria will take the opportunity to hijack certain PIPs to promote infection and play a role in causing disease and increasing virulence. OsPIP1;3 interactions with the bacterial hydrophilic protein *Hpa1* may result in conformational changes of the host cell membrane and contribute to the transport of effector proteins into host cells [46,47].

Furthermore, some proteins do not directly participate in the defense response but form specific microdomains with membrane lipids, thus recruiting receptors to participate in the defense response. Overexpression of NbHIR3.2 or OsHIR3 increased resistance to *Pseudomonas syringae pv*. *tomato* strain DC3000 (*Pst*DC3000) and *Xanthomonas* in tobacco and rice, respectively [48]. OsHIR1 can interact with rice Leucine-Rich Repeat protein 1 (OsLRR1) during pathogen invasion to trigger hypersensitive cell death [49]. Remorins, which are the best characterized marker of plasma membrane microdomains in plants [50], play key roles in plant immunity. For instance, ZmREM6.3 was first reported to be associated with fungal interactions and quantitative disease resistance [51]. In addition, AtREM1.2 interacts with a regulator of PTI and ETI, AtRIN4, indicating that AtREM1.2 can recruit AtRIN4 into membrane microdomains to regulate basal defense [52]. New research proposed that upon perception of PAMPs, Remorin undergoes assembly via intrinsically disordered region mediated oligomerization and recruits Formin into membrane microdomains, which in turn increases actin nucleation [53].

## 3. Dynamics of Membrane Lipids and Proteins Are Regulated by Cytoskeleton

**Figure 1:**
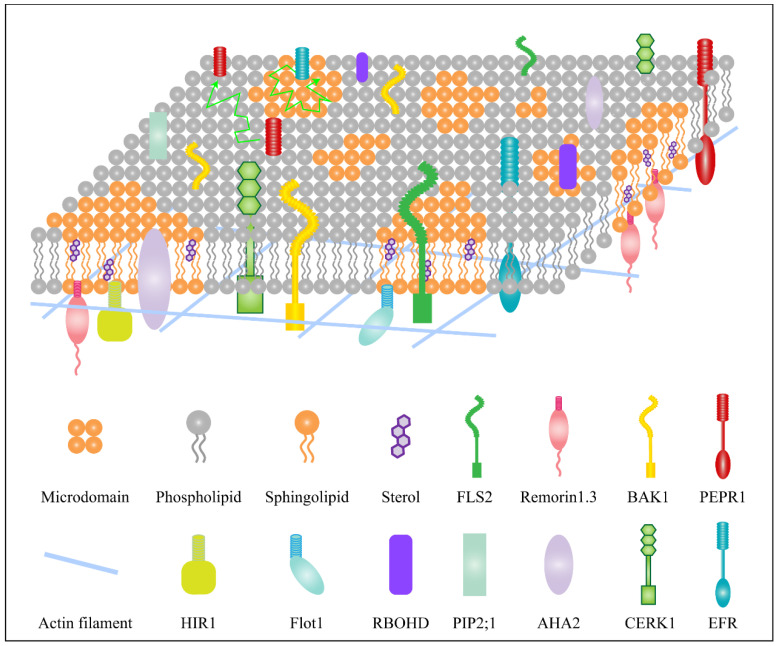
The cytoskeleton is involved in regulating the dynamics and organization of the plasma membrane. The plasma membrane is divided into membrane compartments by the actin cytoskeleton; the membrane compartments’ size is less than 250 nm. The diffusion of PM protein is restricted by the cytoskeleton. The PM proteins have enough free space and kinetic energy to jump to the adjacent compartment. The green line indicates the track route of PM protein movement.

The cytoskeleton is the key factor to control membrane compartmentalization and membrane microdomain dynamics. A number of cytoskeletal proteins is significantly enriched in the DRM fraction, including tubulin, actin, dynamin, myosin, actinin and supervillin, according to biochemical and proteomic studies in mammalian cells [54,55]. On the basis of summarizing the previous work, Kusumi et al. [56] proposed the “picket fence” model to control the lateral diffusion of the membrane (Figure 1). The actin cytoskeleton forms a “fence” which divides the plasma membrane into compartments of 30–250 nm [57]. As a result, raft sizes are restricted to less than the compartment size. Various transmembrane proteins anchored and arranged along the membrane cytoskeleton serve as “pickets”. Diffusion of membrane proteins is hampered by the cytoskeleton, which acts as a steric barrier [58]. For example, the membrane skeleton inhibits the diffusivity of B cell receptors by confining them to compartments [59]. This “picket fence” model is also reported in plants. Several pieces of evidence suggest that the cytoskeleton governs the organization of microdomains in plants.

Moreover, any change in the cytoskeleton organization can affect the distribution or dynamics of microdomain proteins (Figure 1) [58], which further relate to plant immunity responses. A single-particle tracking study demonstrated that the lateral mobility of the microdomain marker protein AtHIR1 was reliant on MT integrity [60]. When pathogen assault occurs, the cytoskeleton, particularly the MTs, limits the dynamic of the AtHIR1, facilitating its oligomerization [60]. Using mass spectroscopy, plasma membrane H^+^-ATPase 2 (AHA2) was identified to be a component of the AtHIR1 complex, which was connected to plant immunity. After treating with cytoskeleton inhibitors Lat B, oryzalin, or sterol-depleting agent MβCD in *Arabidopsis* seedlings, the interactions between AHA2 and HIR1 dramatically diminished. These studies further revealed AtHIR1 may form specific microdomains to recruit AHA2 and to accelerate the formation of the AtHIR1-associated immunological complex in response to pathogens. Similarly, the single molecular dynamics of AtREMs showed significant sensitivity to actin depolymerization [61]. Actin or microtubule depolymerization led to REM1.2 nanodomain size loss and expansion, respectively [27]. In addition, the abundance of AtREM1.2 in DRM decreased when actin and microtubules were destroyed. Meanwhile, a rise in abundance in disordered high-density membrane (DSM) of AtREM1.2 were detected only when actin filaments were damaged [27]. Thereby, cytoskeleton networks are necessary for the dynamic movement of proteins between different membrane phases.

It is noted that following cytoskeleton disruption, phase separations or membrane microdomains would still exist, but their protein makeup would alter. Interestingly, actin and MT play different roles in regulating protein location in microdomains. Using proteomic analysis of cell suspension cultures, proteins which were significantly affected by cytoskeleton destruction were screened. It was discovered that 9 proteins responded significantly to both actin and microtubule disruption, 13 proteins displayed significant relocation in response to oryzalin, while 22 proteins showed significant change abundance distribution between DRM and DSF upon cytochalasin D treatment. In the case of actin inhibitor cytochalasin D treatment, 86% of the proteins in the DRM fraction were reduced; meanwhile, the abundance in the DSM fraction increased [27]. In the majority of instances, although both pharmacological treatments resulted in the depletion of candidate proteins from DRM fractions, only actin disruption with the cytochalasin D treatment resulted in an increase in the abundance of these proteins in the DSM.

In addition to microdomain and microdomain maker proteins, many other proteins have also been reported to be precisely regulated by the cytoskeleton in immunity responses. The signaling pathways connected to receptor activation, mobilization and signal transduction depend largely on the cytoskeleton–PM interface.

PRRs FLS2 and BRI1, interact with BIK1 to form a co-receptor complex, respectively, in response to immunity. In the case of the BRI1–BIK1 complex, after ligand binding, the activated co-receptor complex specifically interacts with the MT network to activate immune signaling [62]. On the contrary, FLS2–BIK1 was reported to interact with actin, which suggests different co-receptor complexes interact with unique cytoskeleton components [63]. It has been discovered that the cytoskeleton has a role in controlling PRR dynamics. A previous study in *Arabidopsis* epidermal cells showed that AtFLS2 lateral mobility reduced after binding with flg22 [64]. After actin or microtubule depolymerization, the AtFLS2 diffusion rate increased in *Arabidopsis* leaf epidermal cells [65]. The dynamics of AtPIP2;1, which plays an important role in the process of stomatal closure in the early stage of immune response triggered by flg22, were primarily governed by the cytoskeleton. Following flg22 treatment, the dwell time of AtPIP2;1 on the PM was longer in guard cells and shorter in subsidiary cells. Further study demonstrated that compared with subsidiary cells, the lateral diffusion and velocity of AtPIP2;1 in guard cells was significantly increased, indicating that guard cells respond to flg22 more sensitively. With Lat B or oryzalin plus flg22 treatment, the velocity and diffusion coefficients of AtPIP2;1 were significantly increased in both guard cells and subsidiary cells, especially the destruction of MTs, demonstrating that MTs are primary regulators of AtPIP2;1 dynamics during stomatal closure in response to flg22 [66].

The dynamic characteristics of membrane proteins are closely related to biological processes. Early research indicates that membrane protein dynamics are important in cellular motility, trafficking and signal transmission [67]. Membrane proteins can participate in complex immune reactions, and the protein dynamics contribute to understanding the mechanism of plant response to pathogens. Hao et al. [68] have reported that flg22 increased the clustering and diffusion coefficient of AtRBOHD particles to facilitate the activation of redox signaling pathways. Su et al. [69] used SPT to monitor the lateral mobility of AtBSK1 at the plasma membrane. After treatment with flg22, the lateral mobility values and diffusion coefficient of AtBSK1 particles were significantly increased, indicating that flg22 induces a change in AtBSK1 dynamics. The activation of AtBSK1 by flg22 resulted in AtBSK1 multimerization, demonstrating that AtBSK1 multimerization was a particular reaction to immunity. BSK1–FLS2 complex localized to Flot1-labeled microdomains in the resting state, but ligand-induced AtBSK1 dissociated from the BSK1–FLS2 complex and translocated to non-microdomain regions. Moreover, the dissociated AtBSK1 interacts with MAPKKK5 to activate downstream immune responses.

## 4. The Cytoskeleton Regulates Endo-Membrane Trafficking of PRRs Involved in Plant Immunity

**Figure 2:**
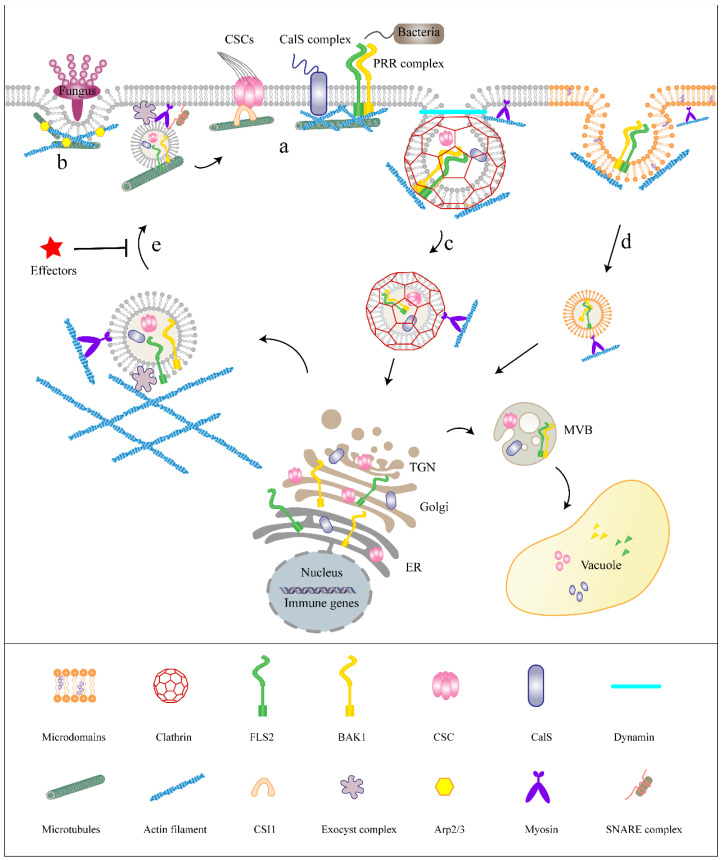
Plant cytoskeleton network regulates endocytic membrane trafficking during the immune response. (a) The plant cytoskeleton offers microenvironments for PRR complexes, CalS complexes and CSCs. (b) The rapid recombination of MF and MT are triggered by the invasion of avirulent fungal strains. (c) The endocytosis of PRR complexes and CW biosynthesis components are predominantly mediated by CME, which require actin cytoskeletons and myosin motors. (d) The turnover of PRR complexes through CIE, which depend on the actin cytoskeleton and myosin motors. (e) The secretion of immune proteins are enhanced under PAMP perception, transporting from TGN to the plasma membrane along the actin cytoskeleton. Pathogen effectors inhibit the secretory trafficking pathways to block defense activation.

The cytoskeleton not only regulates protein dynamics on the plasma membrane but also participates in the intracellular transport of defense molecules in plant immune responses (Figure 2). One of the initial cellular reactions during defense activation is the rapid reorganization of the actin cytoskeleton toward pathogen penetration sites [70]. In order to resist the invasion of pathogenic microorganisms, the precise trafficking of defense molecules to the infection site necessitates both MF and MT. The endo-membrane system and related compartments undergo directional movement, which is largely mediated by the cytoskeleton.

### 4.1. The Role of the Cytoskeleton in Exocytosis of Plant Immune Processes

When immunological signaling is activated, transcription of plasma-membrane-localized immune regulators (i.e., FLS2, EFR, RBOHD and BAK1) enhances. Then, the membrane trafficking system and cytoskeleton are required for the delivery and localization of regulators to the membrane [60]. The transport of PRRs or defensive components to the plasma membrane depends on exocytosis. The cytoskeleton acts as a highway for the trans-Golgi network/early endosome (TGN/EE) or the multivesicular body (MVB) to travel on and is essential for controlling directional secretion [71]. Some research suggests that microscale actin remodeling in secretory hot areas is coupled to nanoscale actin remodeling surrounding exocytosis granules, thereby coordinating the whole exocytosis process [72,73,74]. Through trajectory analyses, it was revealed that the generation of the actin network is usually accompanied by a sharp reduction in the mobility of vesicles, indicating that the actin structure was to tether vesicles at the fusion site [74].

Powdery mildew fungal pathogens may form the haustorium through invagination of the host plasma membrane after invading a leaf cell. Resistance to powdery mildew 8.2 protein (RPW8.2) was highly induced and specifically targeted to the extrahaustorial membrane (EHM), which physically separates the fungal haustorium and host PM. Further experiments showed that the precise aggregation of RPW8.2 in EHM required an intact actin cytoskeleton [75]. With fungal infection in *Arabidopsis*, powdery mildew resistance 4 (PMR4) were also reported to distribute to the EHM via MVB-mediated exocytosis. *Pst* DC3000 increased the biogenesis of MVBs and paramural vesicles that resemble exosomes [76]. Moreover, Thordal-Christensen et al. [77] demonstrated that MVBs were delivered to the penetration site during infection by the *Blumeria graminis f*. *sp*. *Hordei* (*Bgh*). These studies revealed that both MVB biogenesis and exocytosis require the actin cytoskeleton.

Similarly, the delivery of the defense-related protein penetration-resistant 3 (PEN3) in *Arabidopsis* to the host plasma membrane also requires an actin cytoskeleton. When the actin filaments are depolymerized by Lat B or cytochalasin E, the amount of AtPEN3 that accumulates at the site of pathogen penetration is decreased [78]. In addition, the AtPEN1 localization also requires myosin XI. Disrupting myosin by myosin inhibitors or using the myosin XI quadruple knockout mutant prevents pathogen-triggered actin filament reorganization and blocks recruitment of AtPEN1 to the penetration site [15]. Furthermore, actin filaments play a role in the transmission of DAMPs to surrounding cells in order to activate defensive responses [79].

Due to its close ties to the PM and CW, the cytoskeleton can also take part in the dynamics of CW-involved responses to biotic and abiotic stress [80]. Targeting of the PM-CW-localized defensive regulator (cellulose or callose) to the plasma membrane also requires the cytoskeleton. Cellulose is synthesized on the cytoplasmic membrane by cellulose synthase (CESA) complexes (CSCs). Live-cell imaging shows that CESA compartments deliver to the plasma membrane along the actin filaments [81]. When actin filaments were disrupted by using Lat B or using the *act2act7* actin mutant, the transport efficiency of CSCs to the plasma membrane was reduced [81]. It was interesting to note that disrupting actin with Lat B increases CSC’s interaction with cortical MTs, implying a hand-over and/or rivalry between cortical MTs and cortical actin filaments for transport vesicles [82]. A number of studies have reported that MT plays an important role in determining the speed and direction of CSC movement in the plasma membrane. The tracking experiment of fluorescent-protein-labeled CESA proved that CSC showed bidirectional movement along the MT skeleton [83]. Studies of intracellular transport of CSC revealed that some CSC vesicles in the cellular periplasmic reticulum can move along a single microtubule, and the rate of vesicle movement is consistent with the rate of microtubule depolymerization [84]. When MTs were depolymerized with oryzalin, the linear distribution of CSC was destroyed and showed a uniform distribution pattern. Inversely, stabilization of MT with paclitaxel enhanced the linear arrangement of CSC [85]. MT skeletons and motor proteins are considered to slow the transport of organelles or vesicles by anchoring, thereby facilitating the precise secretion of cell wall substances. Furthermore, MT and motor protein Kinesin-4 were involved in the transport of non-cellulose polysaccharide-containing vesicles, which affected the synthesis and properties of plant cell walls [86]. Studies in callose deposition also demonstrated that callose synthase (CalS) sorting in the Golgi and translocating to the cell wall require MF and MT activity [16]. MAMP-induced callose deposition was significantly reduced in *adf4* and *cap-1* cytoskeleton-associated protein mutants compared with the wild type, suggesting that MF regulates callose deposition in PTI immune responses [87,88]. Disruption of MT trafficking severely interferes with apoplast secretion of callose [89,90].

The cytoskeleton network not only plays an active role in promoting the transport of secretory vesicles to fusion sites but can also recruit various exocytosis regulators (i.e., SNARE proteins) to coordinate the final stage of exocytosis. The final stage in exocytosis is SNARE-mediated vesicle docking and fusion at the target membrane. In the *Physcomitrella patens*, Lat B treatment disrupted the distribution of secretory vesicles marked by Vamp72 [91]. Similarly, the distribution of SNARE proteins SYP123, SYP124 and SYP125 was severely affected by Lat B treatment [92]. In the mouse MIN6 cell line, actin filaments are able to directly interact with SNARE proteins. This interaction is weakened by actin filament depolymerization drug treatment, leading to the remodeling of the actin filaments to promote insulin delivery and secretion to the plasma membrane [93]. The analysis of interaction components of the *Arabidopsis* SNARE protein also identified some actin and actin-related proteins [94]. Lastly, Meunier et al. revealed that actin and myosin II surrounding the vesicles work together to promote the release of defense molecules [95]. These studies reveal that the cytoskeleton regulates exocytotic behavior during the anchoring and fusion of secretory vesicles to the plasma membrane, thereby promoting defense molecules to response immune signals.

### 4.2. The Role of the Cytoskeleton in Endocytosis of Plant Immune Processes

The endocytic pathway is the most important trafficking pathway for host immunity. During immune signaling, the turnover of immune proteins in intercellular transport through degradation helps to prevent excessive accumulation of receptor proteins which may cause damage to the plant. These endocytic proteins are recycled back to the plasma membrane indirectly via recycling endosomes or into the vacuole via MVB degradation. For instance, the non-activated FLS2 undergoes constitutive endocytosis between the PM and endosomal compartments in a brefeldin A (BFA)-sensitive manner, which does not require the involvement of BAK1 [62]. Yang et al. [96] showed that selective autophagy provides a degradation pathway for non-activated FLS2 to enter the vacuole. Research has shown that orosomucoids (ORM) as selective autophagy receptors interact with non-activated FLS2 and the autophagy key protein ATG8 to mediate the degradation of FLS2. However, when FLS2 was activated by PAMP, the endocytosis of FLS2 was performed through the BFA-insensitive pathway and required the activity of BAK1 [97]. This was further proved using the method of co-localization with an endocytosis marker protein in tobacco. When treated with flg22, the endocytosis vesicles containing FLS2 were mainly co-localized with the late endosomes marked by ARA7/RabF2b and vacuoles marked by VAMP727 [98]. Similarly, it was also observed in *Arabidopsis* that the activated FLS2 endocytosis vesicles mainly existed in MVB and vacuoles [99]. A series of receptor co-expression and ligand uptake experiments demonstrated that only liganded PRRs were internalized to signal transduction. When epidermal leaf cells were treated with TAMRA-flg22, TAMRA-flg22 was observed to colocalize with FLS2-GFP, suggesting that FLS2 can carry flg22 into vacuoles [100]. The ubiquitin–proteasome system (UPS), as a major intracellular protein degradation mechanism, has also been reported to be involved in the degradation of activated FLS2. Ubiquitin ligases PUB12, and PUB13 polyubiquitinates FLS2, thus promoting flg22-induced FLS2 degradation leading to attenuated FLS2-mediated immune signaling [101]. FLS2 interacting with pathogen effector protein AvrPtoB has also been reported to be degraded by ubiquitination [102]. Similarly, live-cell imaging also proved that PEPR1–GFP and TAMRA–Pep1 complexes formed within seconds; then, AtPep1-PEPR1 were internalized and moved to the vacuole concurrently [99]. The above research shows that ligand-bound PRRs are internalized into endosomes and used to regulate activity during PTI.

The endocytic pathways of plasma membrane proteins include clathrin-mediated endocytosis (CME) and clathrin-independent endocytosis (CIE). Although CIE has been reported to be involved in the endo-membrane trafficking of some membrane proteins [103], most studies of plasma-membrane-localized PRR trafficking focused on CME to a great extent [100]. Activated PRRs can internalize depending on CME to activate downstream signals or to undergo degradation to modulate immune response [100]. For instance, when FLS2 was activated by flg22, FLS2 entered clathrin-coated vesicles (CCVs) through CME, and the late-stage endocytic trafficking of FLS2 regulated defense signals [100]. In the case of clathrin heavy chain deficiency, the endocytosis of FLS2 was significantly inhibited, leading to stomatal closure induced by flg22 being significantly impaired, implying that the endocytosis of FLS2 was positively correlated with stomatal immunity [100]. Dynamins play an essential role in catalyzing the scission and release of CCVs from the PM. In the dynamin-related protein 2B (*drp2b*) mutant, ligand-induced FLS2 endocytosis was significantly decreased, reducing resistance protein expression of the late PTI response [104]. FLS2 endocytosis disruption results in decreased ROS generation in response to flg22 [105]. Similar to the receptor kinases FLS2, CME is not only necessary for endocytosis of PEPR1 under resting conditions but also plays an important role in PEPR1-mediated defense responses. It is reported that AtPep1-induced intracellular accumulation was decreased in clathrin heavy chain 2 (*chc2*) knockout mutants, indicating defective internalization and impaired plant phenotypes [99]. Inducible overexpression of Auxilin2, which was a clathrin coat disintegration factor that suppressed CME, was found to block AtPep1-PEPR1 internalization and damage AtPep1-mediated responses [99].

The specific attachment of actin to the clathrin coat contributes to CME. The CME process begins with the building of the clathrin coat at the plasma membrane, followed by membrane invagination, scission and vesicle uncoating [106]. As clathrin strength decreases, the actin signal increases in accordance with the internalization of the endocytic vesicle [107]. Actin is involved in various endocytic stages such as the polymerization of actin filaments against the plasma membrane providing force for membrane bending and scission [108,109]. A recent study found that all CME stages had both branched and unbranched actin filaments, where most of the endocytic cytoskeleton is branched actin filaments [110]. Combined live-cell imaging and atomic force microscopy indicated that CCP closure was greatly hampered by inhibiting actin polymerization, while disrupting actin depolymerization reduced the formation of CCP [111]. In addition, the cytoskeleton also plays an important role in late CME. By applying situ cryo-electron tomography (cryo-ET) to these cells, actin filaments exist at the base, around and below CCP [110]. Subsequently, actin network expansion promotes vesicle scission and invagination growth.

Given the vital functions that the CME plays in membrane trafficking during pathogen infection, the cytoskeleton may be essential for the coordination of endocytosis in plant immunity. Additional work revealed FLS2 endocytosis was inhibited by the myosin inhibitor 2,3-butanedione monoxime, as well as treatment with Lat B [98]. These results suggest that the ligand-mediated endocytosis of PRR requires the involvement of the actin cytoskeleton.

## 5. Conclusions

The cytoskeleton is a highly dynamic intracellular protein fiber network structure that participates in many important physiological processes such as material transport and signal transduction. Defense responses require the cytoskeleton during intricate crosstalk between PTI and ETI, such as the creation of plasma membrane microdomains, regulation of second messenger production, endocytosis and exocytosis of membrane proteins, the precise targeting of defense proteins to the cell membrane, as well as fortification of the cell wall. Recent developments in advanced imaging techniques, new genomics and structural bioinformatics have improved our knowledge of the dynamic activity of the cytoskeleton. More and more actin-binding proteins (ABPs) have been confirmed to be involved in regulating plant immunity, but new ABPs and their immune regulation mechanisms still need to be further explored. In addition, endocytic trafficking plays an important role in immunity; however, the precise molecular and signaling events that recruit endo-membrane components specifically for immunity-related processes remain unknown. In addition, compared with yeast and animal cells, the regulation mechanism of the cytoskeleton system in endocytic trafficking in plants is not very clear. In conclusion, the regulatory mechanism of the cytoskeleton in the process of plant immunity is very complex. Future work should focus more on exploring the fine interactions between the cytoskeleton and membrane systems that will help us understand the biological functions of the cytoskeleton in plant innate immunity.

## Data Availability

Not applicable.
